# Supplementary material for: Mitochondrial One-Carbon Metabolism is Required for TGF-β-Induced Glycine Synthesis and Collagen Protein Production
Source: bioRxiv. 2023 Nov 7:2023.11.07.566074. Preprint. [Version 1] doi: 10.1101/2023.11.07.566074 (PMC10659399; doi:10.1101/2023.11.07.566074)
Supplement: 1 [file NIHPP2023.11.07.566074V1-supplement-1.pdf]

bioRxiv preprint doi: https://doi.org/10.1101/2023.11.07.566074; this version posted November 7, 2023. The copyright holder for this preprint (which was not certified by peer review) is the author/funder, who has granted bioRxiv a license to display the preprint in perpetuity. It is made available under aCC-BY 4.0 International license.

SUPPLEMENTARY FIGURE LEGENDS

Figure S1. Mitochondrial one-carbon enzymes are induced by TGF-β. (A-D) Quantification of relative (A) MTHFD2, (B) ALDH1L2, (C) MTHFD1L, and (D) MTHFD1 protein levels after treatment of normal human lung fibroblasts (NHLFs) treated with TGF-β for the indicated intervals. **(E-H)** Quantification of relative (E) MTHFD2, (F) ALDH1L2, (G) MTHFD1L, and (H) MTHFD1 protein levels after treatment of NHLFs transfected with siRNA targeting ATF4 or nontargeting siRNA. Cells were treated with TGF-β for the indicated intervals. **(I-L)** Quantification of relative (I) MTHFD2, (J) ALDH1L2, (K) MTHFD1L, and (L) MTHFD1 protein levels after treatment of NHLFs with TGF-β for the indicated intervals in the presence or absence of Rapalink-1. Bar graphs

bioRxiv preprint doi: https://doi.org/10.1101/2023.11.07.566074; this version posted November 7, 2023. The copyright holder for this preprint (which was not certified by peer review) is the author/funder, who has granted bioRxiv a license to display the preprint in perpetuity. It is made available under aCC-BY 4.0 International license.

represent mean ± SEM, n=3 independent experimental replicates. **P*<0.05, ***P*<0.01, ****P*<0.001.

Figure S2. Mitochondrial one-carbon metabolism is required for TGF-β-induced collagen protein production. (A-B) Western blot analysis of collagen and α-smooth muscle actin (α-SMA) protein expression in normal human lung fibroblasts (NHLFs) transfected with siRNAs targeting *MTHFD2* or nontargeting siRNA. Cells were treated with TGF-β for the indicated intervals in the presence of the indicated doses of formate. **(C)** Western blot analysis of collagen and α-SMA protein expression in NHLFs treated TGF-β for the indicated intervals in the presence of the indicated doses of methotrexate (MTX). **(D-E)** Western blot analysis of collagen and α-SMA protein expression in NHLFs transfected with siRNAs targeting *MTHFD2* or nontargeting siRNA. Cells were treated with TGF-β for the indicated intervals in the presence of the indicated doses of 5-methyl-tetrahydrofolate (mTHF).

Figure S3. MTHFD2 is required for increased cellular glycine levels downstream of TGF-β. (A) Schematic representation of metabolite labeling downstream of ^{13}C2-Glycine. Normal human lung fibroblasts (NHLFs) were transfected with siRNA targeting *MTHFD2* or nontargeting siRNA. Cells were labeled with ^{13}C2-Glycine and treated with TGF-β for 48 hours or left untreated. **(B)** Analysis of cellular glycine after labeling with ^{13}C2-Glycine in NHLFs treated with TGF-β or left untreated. **(C)** Relative total glycine levels from (B). **(D)** Relative levels of M+0 (*de novo* synthesized) glycine from (B). **(E)** Analysis of cellular serine after labeling with ^{13}C2-Glycine in NHLFs treated with TGF-β or left untreated. **(F)** Relative total serine levels from (E). **(G)** Relative levels of M+2 serine from (E). Bar graphs represent mean ± SEM, n=3. **P*<0.05, ***P*<0.01, ****P*<0.001.

Figure S4. SHMT2 is required for increased cellular glycine levels downstream of TGF-β. Normal human lung fibroblasts (NHLFs) were transfected with siRNA targeting *SHMT2* or nontargeting siRNA. Cells were labeled with ^{13}C2-Glycine and treated with TGF-β for 48 hours or

bioRxiv preprint doi: https://doi.org/10.1101/2023.11.07.566074; this version posted November 7, 2023. The copyright holder for this preprint (which was not certified by peer review) is the author/funder, who has granted bioRxiv a license to display the preprint in perpetuity. It is made available under aCC-BY 4.0 International license.

left untreated. **(A)** Analysis of cellular glycine after labeling with ^{13}C2-Glycine in NHLFs treated with TGF-β or left untreated. **(B)** Relative total glycine levels from (A). **(C)** Relative levels of M+0 (*de novo* synthesized) glycine from (A). **(D)** Analysis of cellular serine after labeling with ^{13}C2-Glycine in NHLFs treated with TGF-β or left untreated. **(E)** Relative total serine levels from (D). **(F)** Relative levels of M+2 serine from (D). Bar graphs represent mean ± SEM, n=3. **P*<0.05, ***P*<0.01, ****P*<0.001.

Figure S5. Analysis of cellular metabolite levels in *MTHFD2* knockdown human lung fibroblasts. Normal human lung fibroblasts (NHLFs) were transfected with siRNA targeting *MTHFD2* or nontargeting siRNA. Cells were labeled with 2,3,3-D3-Serine and treated with TGF-β for 48 hours or left untreated. **(A)** Relative total glycine levels from Figure 3B. **(B)** Relative total serine levels from Figure 3D. **(C)** Relative total proline levels from Figure 3F. Bar graphs represent mean ± SEM, n=3. **P*<0.05, ***P*<0.01, ****P*<0.001.

Figure S6. Analysis of cellular metabolite levels in *ALDH1L2* knockdown human lung fibroblasts. Normal human lung fibroblasts (NHLFs) were transfected with siRNA targeting *ALDH1L2* or nontargeting siRNA. Cells were labeled with 2,3,3-D3-Serine and treated with TGF-β for 48 hours or left untreated. **(A)** Relative total glycine levels from Figure 4A. **(B)** Relative levels of M+1 glycine from Figure 4A. **(C)** Relative total serine levels from Figure 4B. **(D)** Relative levels of M+1 and M+2 serine from Figure 4B. **(E)** Relative total proline levels from Figure 4C. **(F)** Relative levels of M+1 proline from Figure 4C. Bar graphs represent mean ± SEM, n=3. **P*<0.05, ***P*<0.01, ****P*<0.001.

Figure S7. Analysis of cellular metabolite levels in *MTHFD1L* knockdown human lung fibroblasts. Normal human lung fibroblasts (NHLFs) were transfected with siRNA targeting *MTHFD1L* or nontargeting siRNA. Cells were labeled with 2,3,3-D3-Serine and treated with TGF-β for 48 hours or left untreated. **(A)** Relative total glycine levels from Figure 4E. **(B)** Relative levels of M+1 glycine from Figure 4E. **(C)** Relative total serine levels from Figure 4F. **(D)** Relative levels

bioRxiv preprint doi: https://doi.org/10.1101/2023.11.07.566074; this version posted November 7, 2023. The copyright holder for this preprint (which was not certified by peer review) is the author/funder, who has granted bioRxiv a license to display the preprint in perpetuity. It is made available under aCC-BY 4.0 International license.

of M+1 and M+2 serine from Figure 4F. **(E)** Relative total proline levels from Figure 4G. **(F)** Relative levels of M+1 proline from Figure 4G. Bar graphs represent mean ± SEM, n=3. **P*<0.05, ***P*<0.01, ****P*<0.001.

Figure S8. Analysis of cellular metabolite levels in human lung fibroblasts with knockdown of both *ALDH1L2* and *MTHFD1L*. Normal human lung fibroblasts (NHLFs) were transfected with siRNA targeting both *ALDH1L2* and *MTHFD1L* or nontargeting siRNA. Cells were labeled with 2,3,3-D3-Serine and treated with TGF-β for 48 hours or left untreated. **(A)** Relative total glycine levels from Figure 4I. **(B)** Relative levels of M+1 glycine from Figure 4I. **(C)** Relative total serine levels from Figure 4J. **(D)** Relative levels of M+1 and M+2 serine from Figure 4J. **(E)** Relative total proline levels from Figure 4K. **(F)** Relative levels of M+1 proline from Figure 4K. Bar graphs represent mean ± SEM, n=3. **P*<0.05, ***P*<0.01, ****P*<0.001.

Figure S9. *MTHFD1* is not required for increased cellular glycine levels downstream of TGF-β. Normal human lung fibroblasts (NHLFs) were transfected with siRNA targeting *MTHFD1* or nontargeting siRNA. Cells were labeled with 2,3,3-D3-Serine and treated with TGF-β for 48 hours or left untreated. **(A)** Analysis of cellular glycine after labeling with 2,3,3-D3-Serine in NHLFs treated with TGF-β or left untreated. **(B)** Relative total glycine levels from (A). **(C)** Relative levels of M+1 glycine from (A). **(D)** Analysis of cellular serine after labeling with 2,3,3-D3-Serine in NHLFs treated with TGF-β or left untreated. **(E)** Relative total serine levels from (D). **(F)** Relative levels of M+1 and M+2 serine from (D). **(G)** Analysis of cellular proline after labeling with 2,3,3-D3-Serine in NHLFs treated with TGF-β or left untreated. **(H)** Relative total serine levels from (G). **(I)** Relative levels of M+1 and M+2 serine from (G). Bar graphs represent mean ± SEM, n=3. **P*<0.05, ***P*<0.01, ****P*<0.001.

Figure S10. Pharmacologic inhibition of MTHFD2 inhibits fibrotic responses. (A) Relative total glycine levels from Figure 5E. **(B)** Relative levels of M+1 glycine from Figure 5E. **(C)** Relative total serine levels from Figure 5F. **(D)** Relative levels of M+1 and M+2 serine from Figure 5F. **(E)**

bioRxiv preprint doi: https://doi.org/10.1101/2023.11.07.566074; this version posted November 7, 2023. The copyright holder for this preprint (which was not certified by peer review) is the author/funder, who has granted bioRxiv a license to display the preprint in perpetuity. It is made available under aCC-BY 4.0 International license.

Relative total proline levels from Figure 5G. **(F)** Relative levels of M+1 proline from Figure 5G. Bar graphs represent mean ± SEM, n=3. **(G)** Representative transverse microCT images of mice prior to bleomycin instillation (Day 0), 7 days after bleomycin instillation (Day 7), and 14 days after bleomycin instillation (Day 14). Mice began receiving DS18561882 (125 mg/kg) or vehicle 8 days after bleomycin instillation. **(H)** Percent fibrotic area of lung from mice 21 days after bleomycin instillation. Mice received either vehicle or DS18561882 beginning on day 8. Bar graphs represent mean ± SEM, n=5 independent mice per condition. *P<0.05, **P<0.01, ***P<0.001.

Figure S1

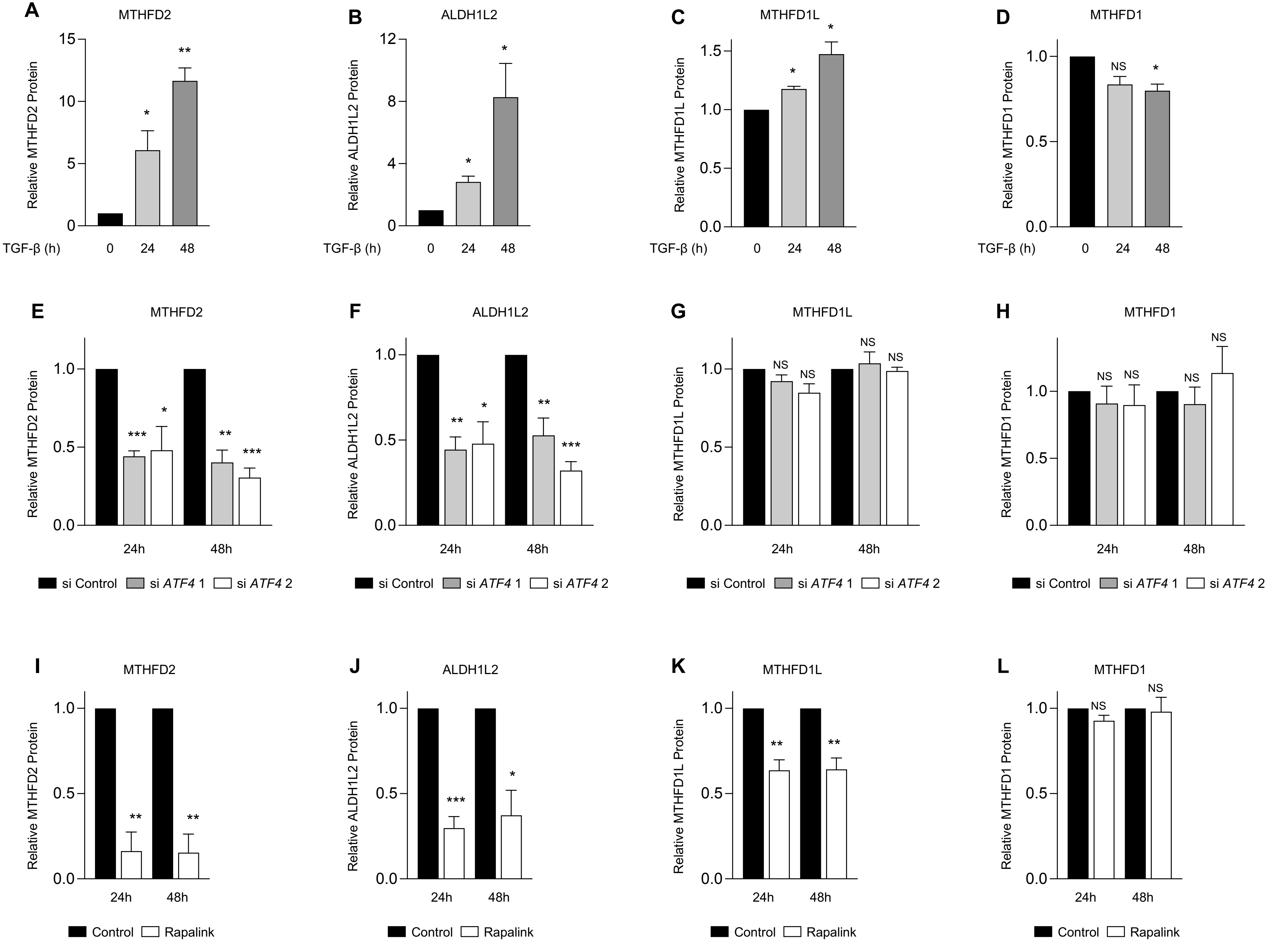

A
MTHFD2
Relative MTHFD2 Protein
TGF-β (h)
0
24
48
B
ALDH1L2
Relative ALDH1L2 Protein
C
MTHFD1L
Relative MTHFD1L Protein
D
MTHFD1
Relative MTHFD1 Protein
NS
E
F
G
H
24h
48h
si Control
si ATF4 1
si ATF4 2
I
J
K
L
Control
Rapalink

Figure S2

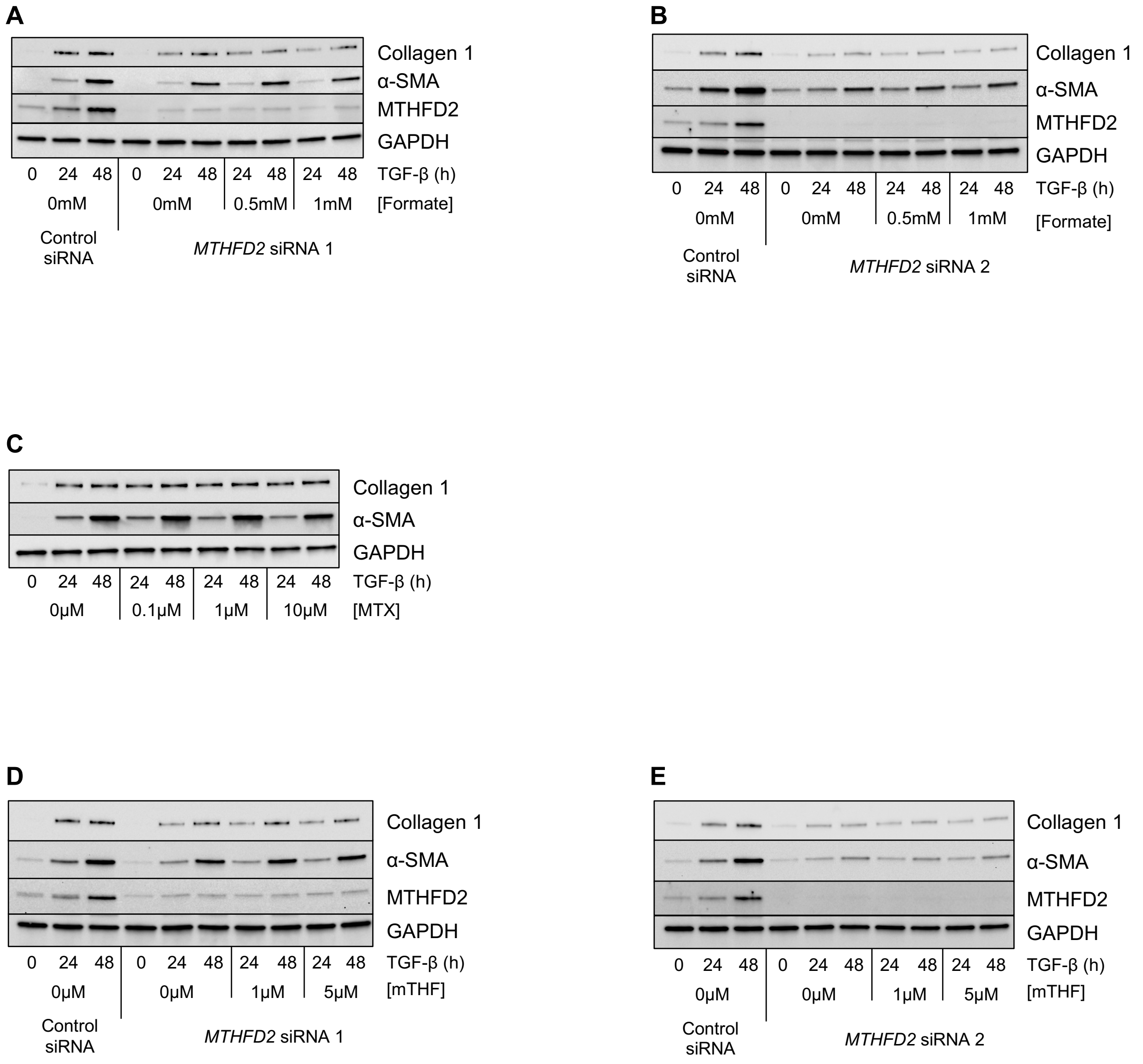
A
Collagen 1
α-SMA
MTHFD2
GAPDH
0 24 48 0 24 48 24 48 24 48 TGF-β (h)
0mM 0mM 0.5mM 1mM [Formate]
Control siRNA
MTHFD2 siRNA 1
B
Collagen 1
α-SMA
MTHFD2
GAPDH
0 24 48 0 24 48 24 48 24 48 TGF-β (h)
0mM 0mM 0.5mM 1mM [Formate]
Control siRNA
MTHFD2 siRNA 2
C
Collagen 1
α-SMA
GAPDH
0 24 48 24 48 24 48 24 48 TGF-β (h)
0µM 0.1µM 1µM 10µM [MTX]
D
Collagen 1
α-SMA
MTHFD2
GAPDH
0 24 48 0 24 48 24 48 24 48 TGF-β (h)
0µM 0µM 1µM 5µM [mTHF]
Control siRNA
MTHFD2 siRNA 1
E
Collagen 1
α-SMA
MTHFD2
GAPDH
0 24 48 0 24 48 24 48 24 48 TGF-β (h)
0µM 0µM 1µM 5µM [mTHF]
Control siRNA
MTHFD2 siRNA 2

Figure S3

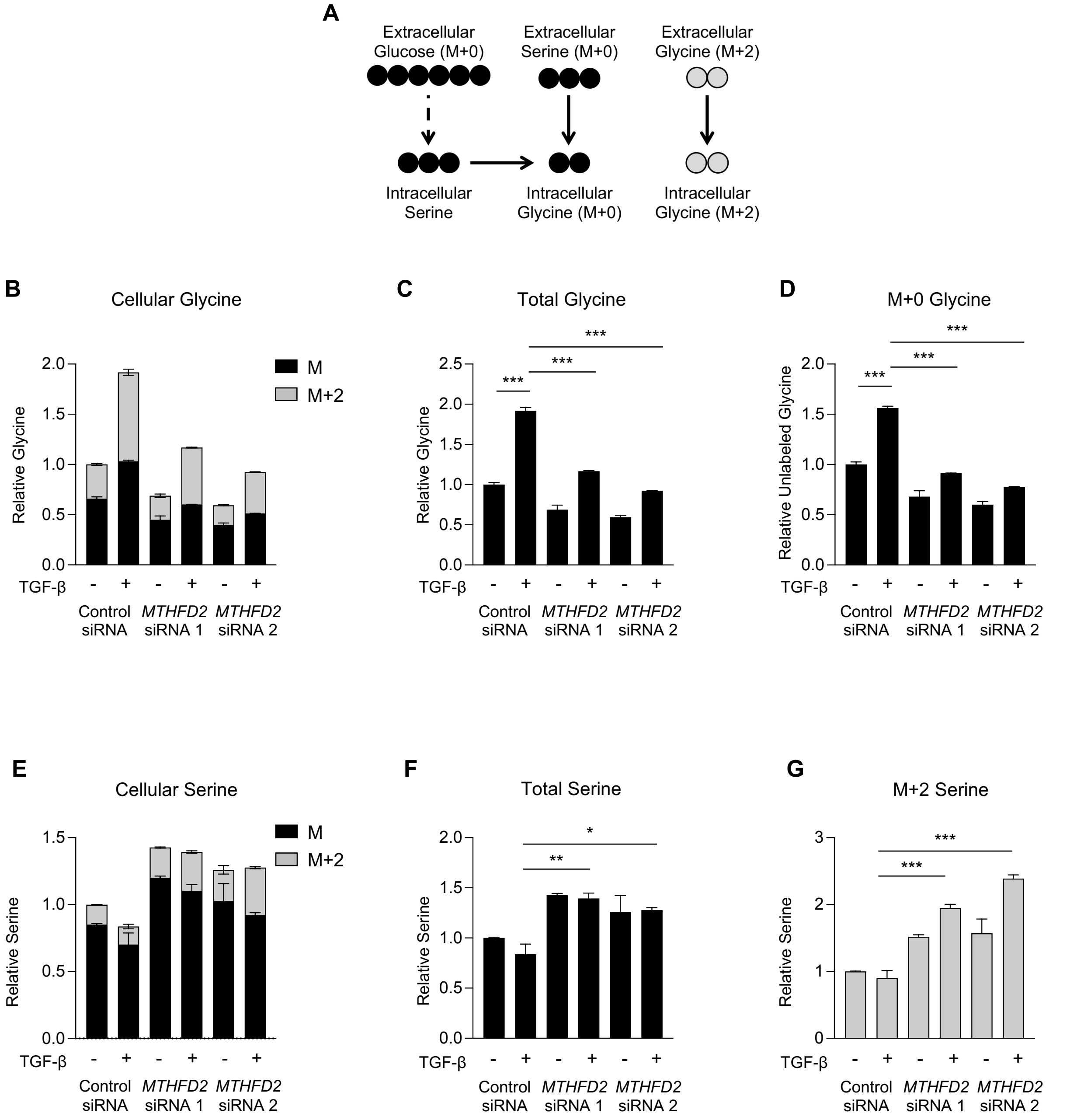
A
Extracellular Glucose (M+0)
Extracellular Serine (M+0)
Extracellular Glycine (M+2)
Intracellular Serine
Intracellular Glycine (M+0)
Intracellular Glycine (M+2)
B
Cellular Glycine
Relative Glycine
M
M+2
C
Total Glycine
Relative Glycine
D
M+0 Glycine
Relative Unlabeled Glycine
E
Cellular Serine
Relative Serine
F
Total Serine
Relative Serine
G
M+2 Serine
Relative Serine
TGF-β
Control siRNA
MTHFD2 siRNA 1
MTHFD2 siRNA 2

Figure S4

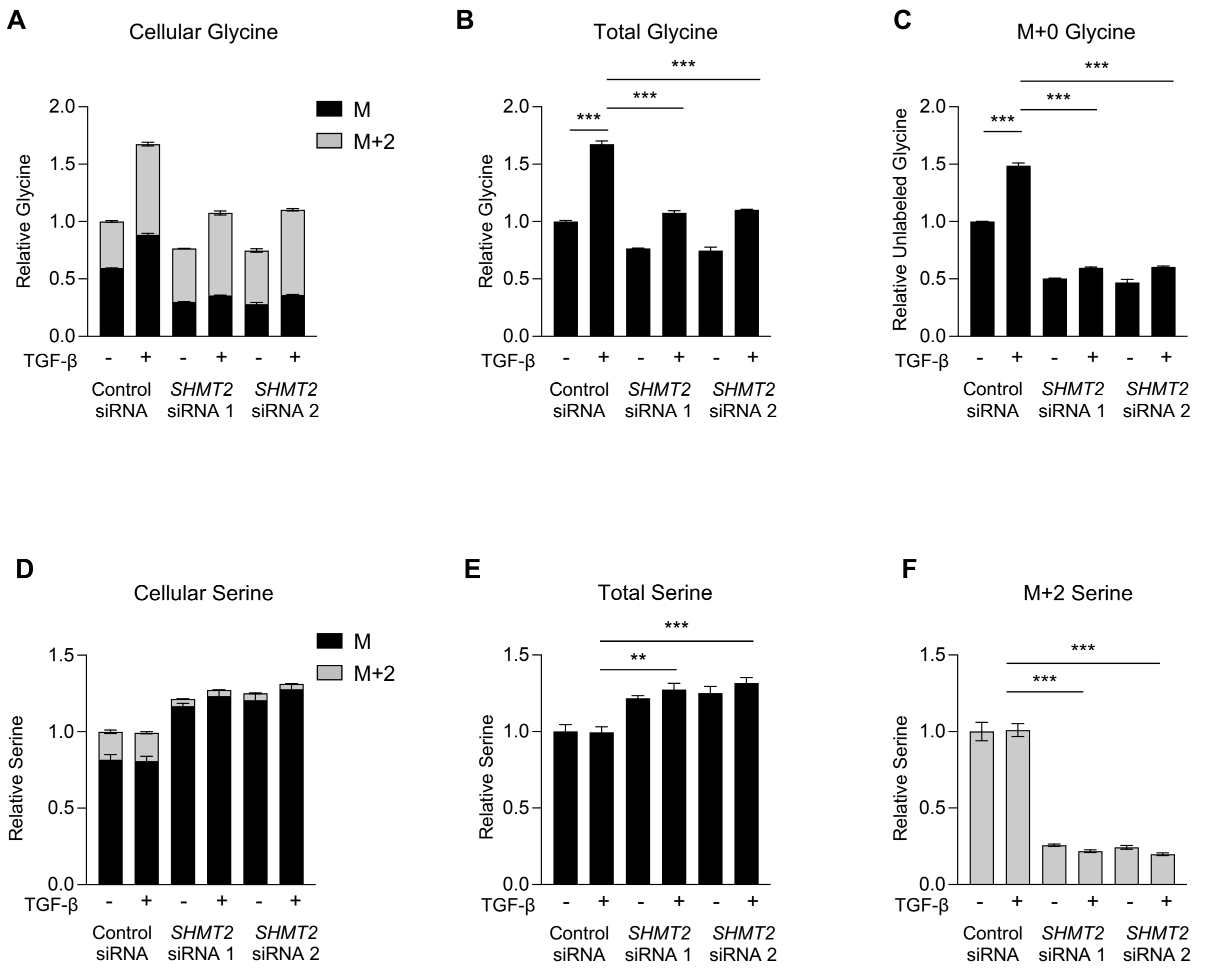
A
Cellular Glycine
Relative Glycine
M
M+2
TGF-β
Control siRNA
SHMT2 siRNA 1
SHMT2 siRNA 2
B
Total Glycine
Relative Glycine
C
M+0 Glycine
Relative Unlabeled Glycine
D
Cellular Serine
Relative Serine
E
Total Serine
Relative Serine
F
M+2 Serine
Relative Serine

Figure S5

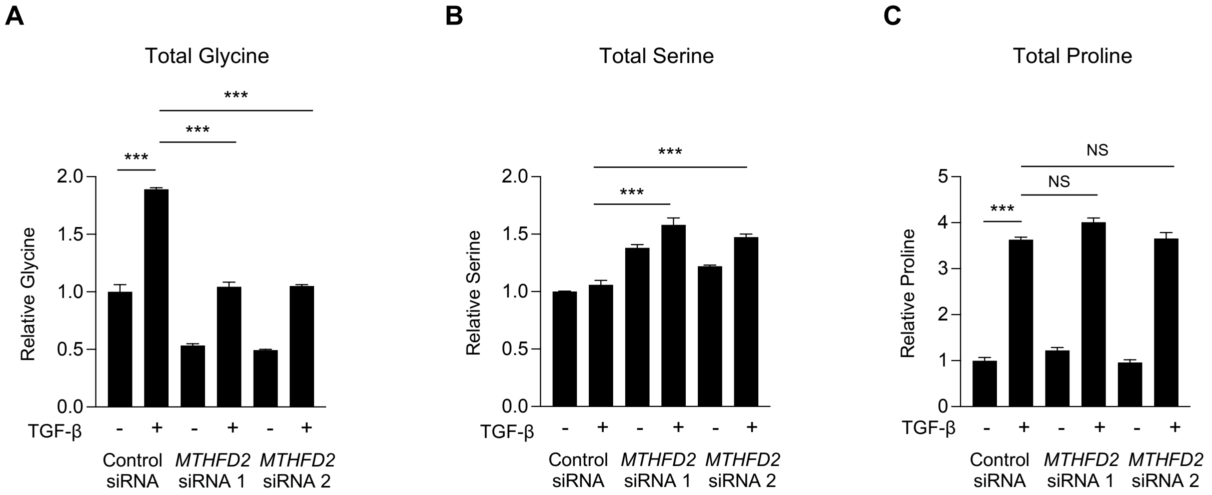
A
Total Glycine
***
***
***
Relative Glycine
2.0
1.5
1.0
0.5
0.0
TGF-β
- + - + - +
Control siRNA
MTHFD2 siRNA 1
MTHFD2 siRNA 2
B
Total Serine
***
***
Relative Serine
2.0
1.5
1.0
0.5
0.0
TGF-β
- + - + - +
Control siRNA
MTHFD2 siRNA 1
MTHFD2 siRNA 2
C
Total Proline
NS
NS
***
Relative Proline
5
4
3
2
1
0
TGF-β
- + - + - +
Control siRNA
MTHFD2 siRNA 1
MTHFD2 siRNA 2

Figure S6

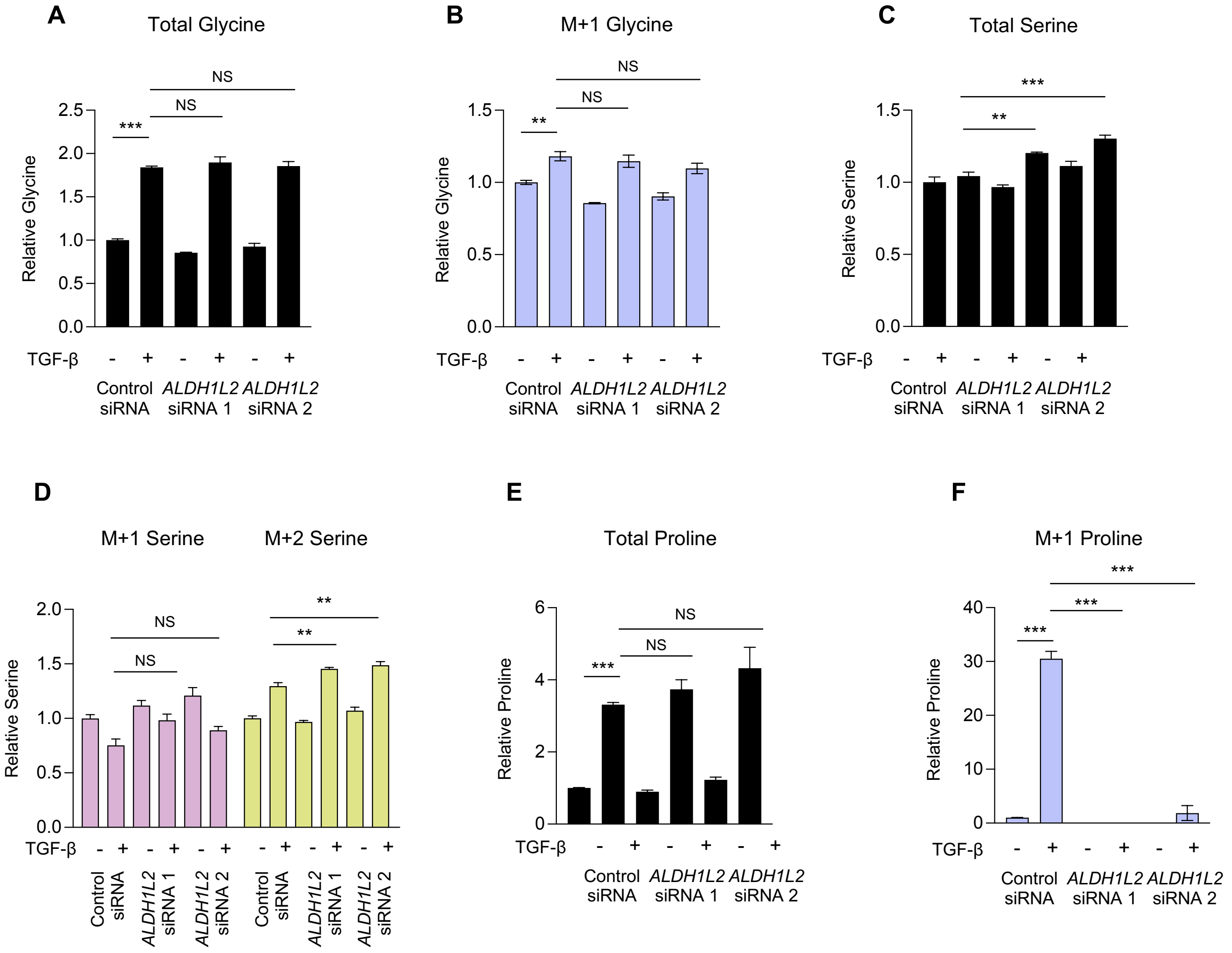
A
Total Glycine
Relative Glycine
TGF-β
Control siRNA
ALDH1L2 siRNA 1
ALDH1L2 siRNA 2
B
M+1 Glycine
C
Total Serine
Relative Serine
D
M+1 Serine
M+2 Serine
E
Total Proline
Relative Proline
F
M+1 Proline
NS
***
**

Figure S7

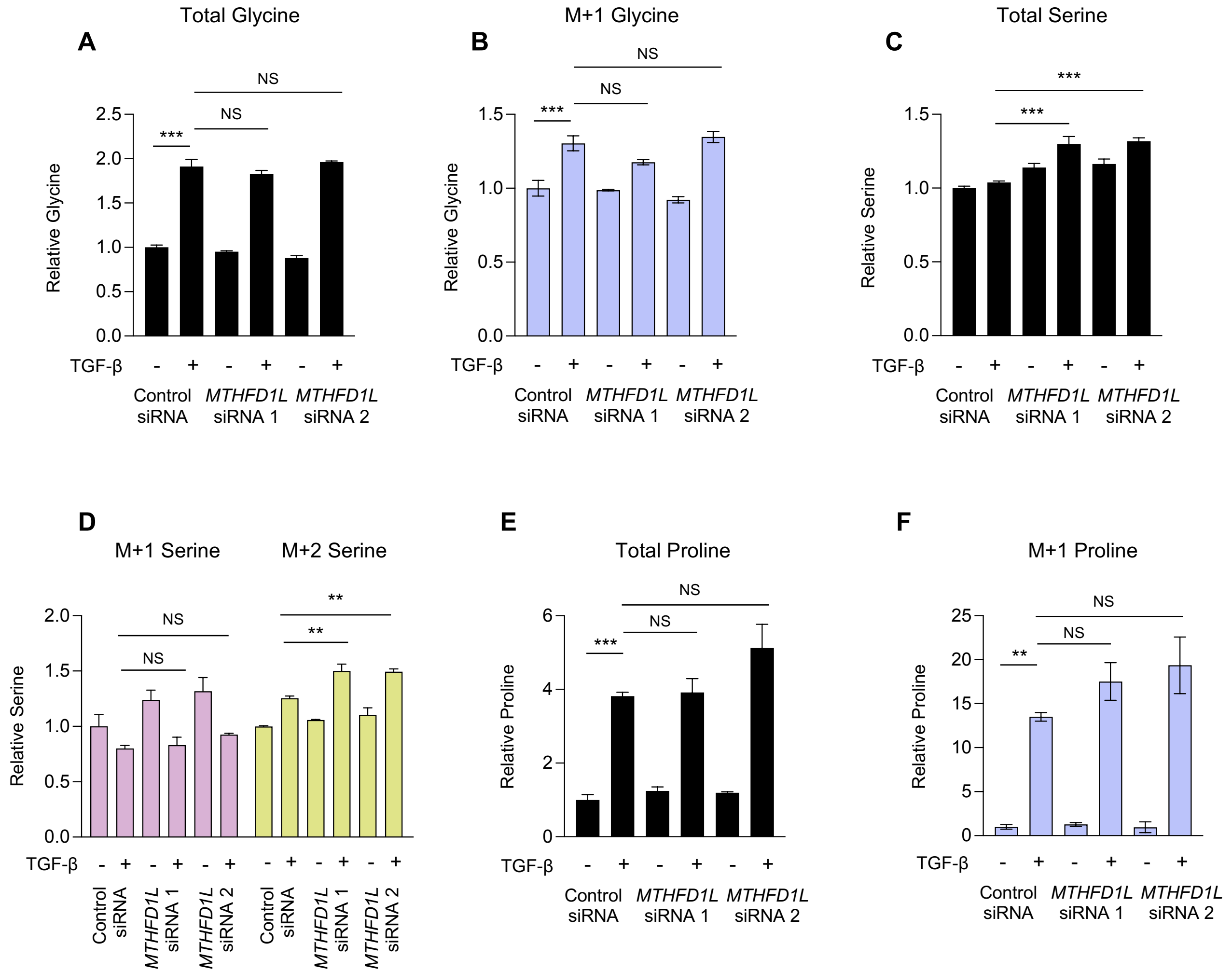
A
Total Glycine
Relative Glycine
2.5
2.0
1.5
1.0
0.5
0.0
***
NS
NS
TGF-β
- + - + - +
Control siRNA
MTHFD1L siRNA 1
MTHFD1L siRNA 2
B
M+1 Glycine
Relative Glycine
1.5
1.0
0.5
0.0
***
NS
NS
C
Total Serine
Relative Serine
1.5
1.0
0.5
0.0
***
***
D
M+1 Serine
M+2 Serine
Relative Serine
2.0
1.5
1.0
0.5
0.0
NS
NS
**
**
E
Total Proline
Relative Proline
6
4
2
0
***
NS
NS
F
M+1 Proline
Relative Proline
25
20
15
10
5
0
**
NS
NS

Figure S8

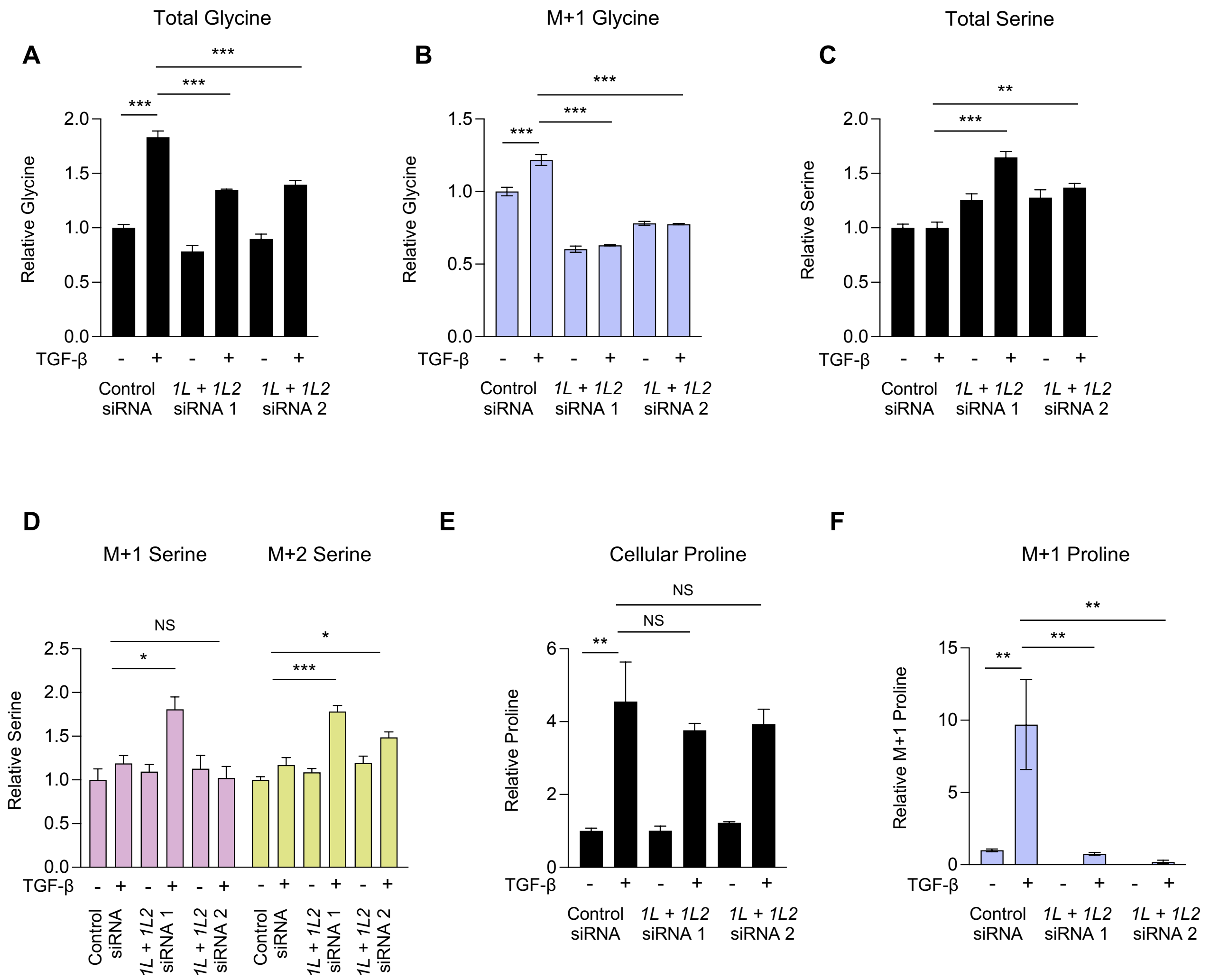
Total Glycine
M+1 Glycine
Total Serine
A
B
C
Relative Glycine
Relative Serine
TGF-β
Control siRNA
1L + 1L2 siRNA 1
1L + 1L2 siRNA 2
D
E
F
M+1 Serine
M+2 Serine
Cellular Proline
M+1 Proline
Relative Proline
Relative M+1 Proline
NS
***
**
*

Figure S9

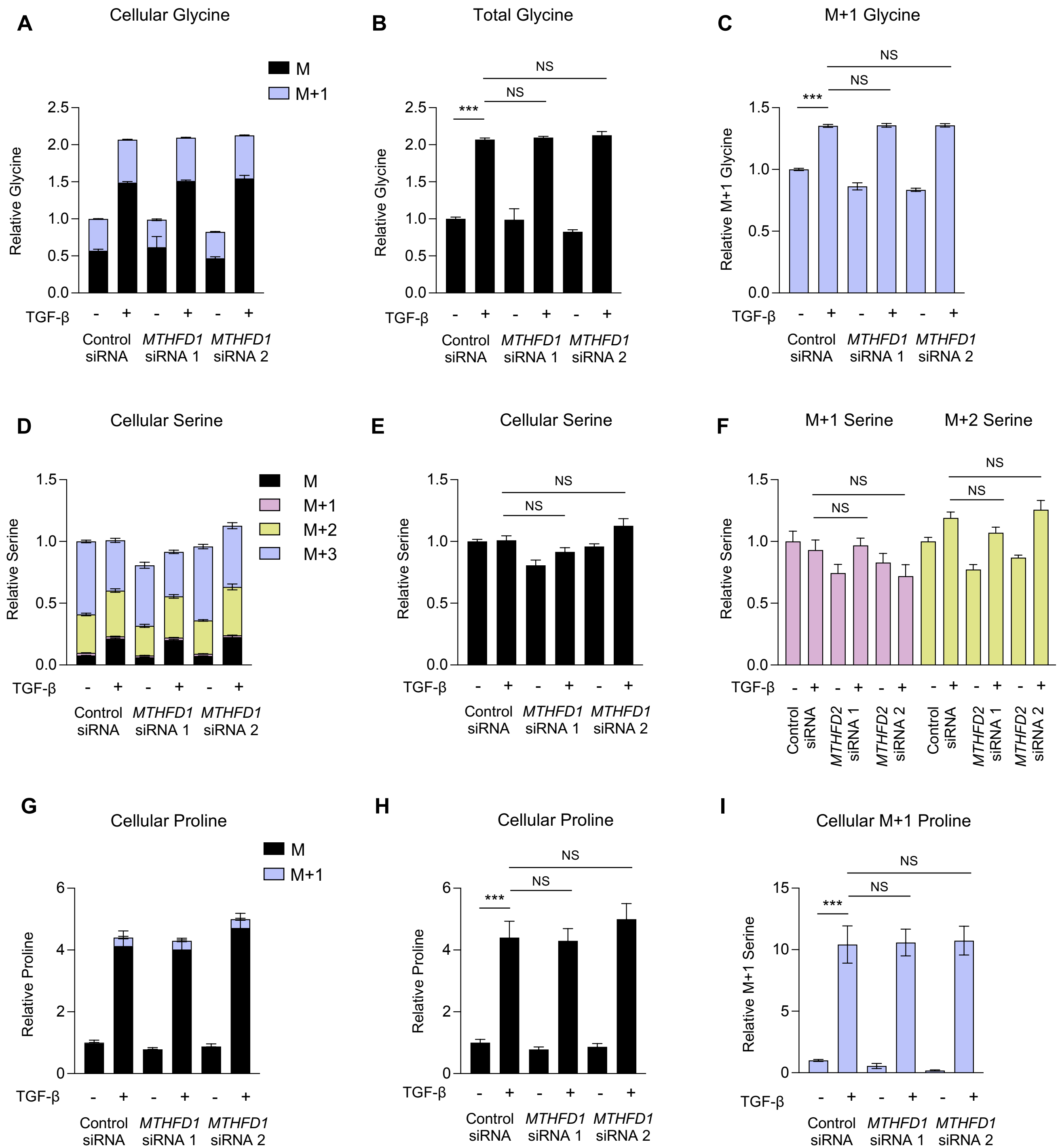
A
Cellular Glycine
M
M+1
Relative Glycine
TGF-β
Control siRNA
MTHFD1 siRNA 1
MTHFD1 siRNA 2
B
Total Glycine
***
NS
NS
Relative Glycine
C
M+1 Glycine
Relative M+1 Glycine
D
Cellular Serine
M
M+1
M+2
M+3
Relative Serine
E
Cellular Serine
F
M+1 Serine
M+2 Serine
MTHFD2 siRNA 1
MTHFD2 siRNA 2
G
Cellular Proline
Relative Proline
H
Cellular Proline
I
Cellular M+1 Proline
Relative M+1 Serine

Figure S10

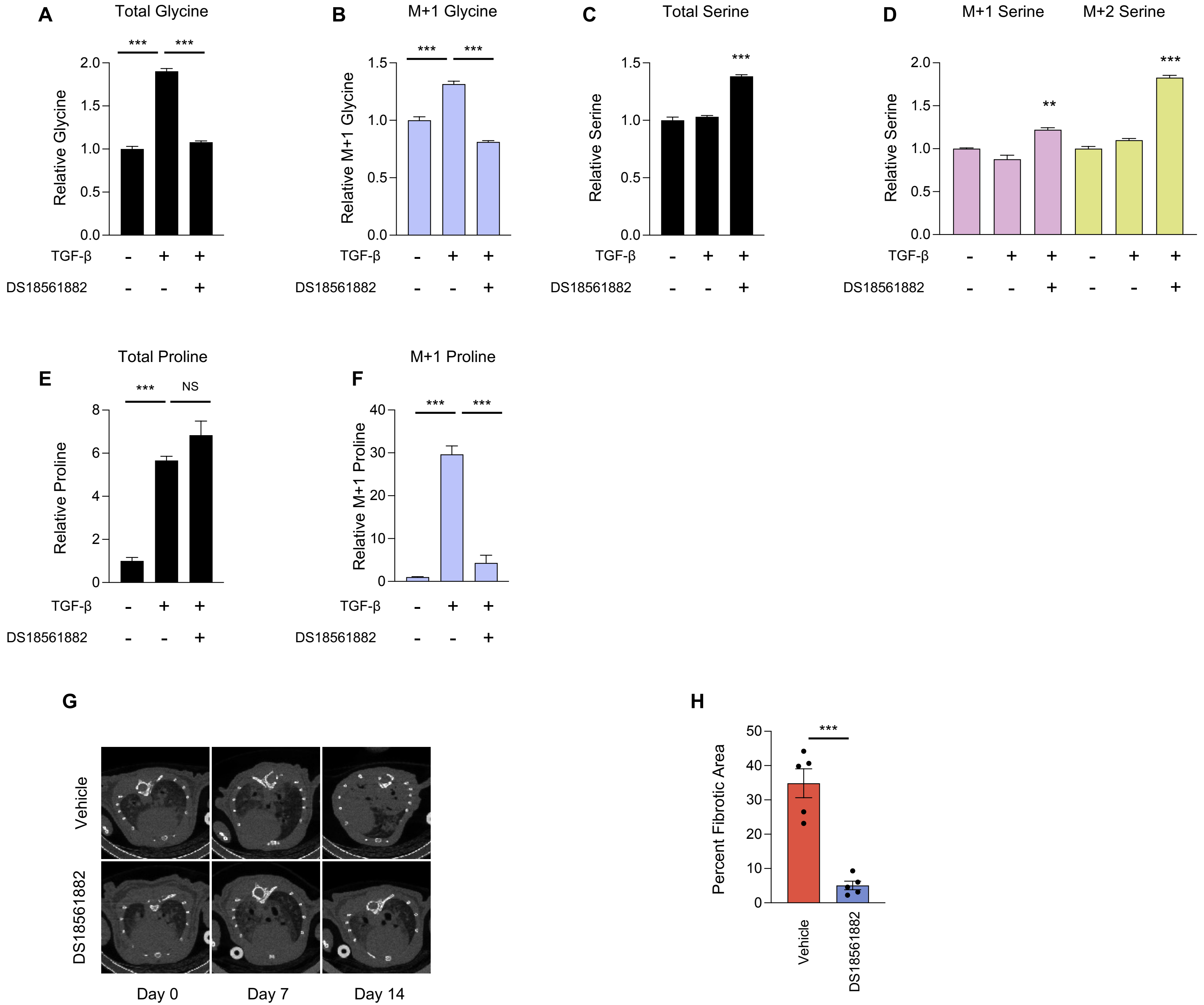
A
Total Glycine
Relative Glycine
***
***
TGF-β - + +
DS18561882 - - +
B
M+1 Glycine
Relative M+1 Glycine
***
***
TGF-β - + +
DS18561882 - - +
C
Total Serine
Relative Serine
***
TGF-β - + +
DS18561882 - - +
D
M+1 Serine
M+2 Serine
Relative Serine
**
***
TGF-β - + + - + +
DS18561882 - - + - - +
E
Total Proline
Relative Proline
***
NS
TGF-β - + +
DS18561882 - - +
F
M+1 Proline
Relative M+1 Proline
***
***
TGF-β - + +
DS18561882 - - +
G
Vehicle
DS18561882
Day 0
Day 7
Day 14
H
Percent Fibrotic Area
***
Vehicle
DS18561882
